# Mineral bone disorder in children with chronic kidney disease: Data from the KNOW-Ped CKD (Korean cohort study for outcome in patients with pediatric chronic kidney disease) study

**DOI:** 10.3389/fped.2023.994979

**Published:** 2023-02-17

**Authors:** Jiwon Jung, Keum Hwa Lee, Eujin Park, Young Seo Park, Hee Gyung Kang, Yo Han Ahn, Il-Soo Ha, Seong Heon Kim, Heeyeon Cho, Kyoung Hee Han, Min Hyun Cho, Hyun Jin Choi, Joo Hoon Lee, Jae Il Shin

**Affiliations:** ^1^ Department of Pediatrics, Asan Medical Center Children's Hospital, Ulsan University, College of Medicine, Seoul, Republic of Korea; ^2^ Department of Pediatrics, Severance Children's Hospital, College of Medicine, University of Yonsei, Seoul, Republic of Korea; ^3^ Department of Pediatrics, Hallym University Kangnam Sacred Heart Hospital, Seoul, Republic of Korea; ^4^ Department of Pediatrics, Seoul National University Children's Hospital, College of Medicine, Seoul National University, Seoul, Republic of Korea; ^5^ Department of Pediatrics, Samsung Medical Center, School of Medicine, Sungkyunkwan University, Seoul, Republic of Korea; ^6^ Department of Pediatrics, School of Medicine, Jeju National University, Jeju, Republic of Korea; ^7^ Department of Pediatrics, School of Medicine, Kyungpook National University, Daegu, Republic of Korea; ^8^ National Institute of Food and Drug Safety Evaluation, Ministry of Food and Drug Safety, Cheongju, Republic of Korea; ^9^ Institute of Kidney Disease Research, College of Medicine, Yonsei University, Seoul, Republic of Korea

**Keywords:** mineral bone disorder, children, hyperphosphatemia, hyperparathyroidism, chronic kidney disease, bone densitometry

## Abstract

**Background:**

Children with chronic kidney disease (CKD) are at high risk of mineral bone disorder (MBD), which leads to fractures, growth retardation, and cardiovascular disease. We aimed to comprehensively understand the relationship between renal function and factors related to MBD and evaluate the prevalence and distribution characteristics of MBD, specifically among Korean patients from the KNOW-PedCKD cohort.

**Methods:**

From the baseline data of the KNOW-PedCKD cohort, we examined the prevalence and distribution of MBD in 431 Korean pediatric CKD patients, including the level of corrected total calcium, serum phosphate, serum alkaline phosphatase, serum intact parathyroid hormone (iPTH), fibroblast growth factor 23 (FGF-23), serum vitamin D, fractional excretion of phosphate (FEP), and bone densitometry Z-scores.

**Results:**

The median serum calcium level remained relatively normal regardless of the CKD stage. The levels of 1,25-dihydroxy vitamin D, urine calcium-to-creatinine ratio, and bone densitometry Z-score significantly decreased with advancing CKD stage, while those of serum phosphate, FGF-23, and FEP significantly increased with CKD stage. The prevalence of hyperphosphatemia (17.4%, 23.7%, and 41.2% from CKD stages 3b, 4, and 5, respectively) and hyperparathyroidism (37.3%, 57.4%, 55.3%, and 52.9% from CKD stages 3a, 3b, 4, and 5, respectively) significantly increased with the CKD stage. Prescriptions of medications, such as calcium supplements (39.1%, 42.1%, 82.4%), phosphate binders (39.1%, 43.4%, 82.4%), and active vitamin D (21.7%, 44.7%, and 64.7%) significantly increased with CKD stage 3b, 4, and 5, respectively.

**Conclusions:**

The results demonstrated the prevalence and relationship of abnormal mineral metabolism and bone growth according to CKD stage in Korean pediatric CKD patients for the first time.

## Introduction

Mineral bone disorder (MBD) represents a systemic disorder manifested by either one or a combination of abnormal mineral metabolisms, abnormal bone growth or strength, making bone more vulnerable to fracture and extra-skeletal calcification, all of which are closely interrelated and contribute to the morbidity and mortality of patients with chronic kidney disease (CKD), including those with cardiovascular disease (1). In 2006, the Kidney Disease: Improving Global Outcomes (KDIGO) working group addressed this revised terminology, replacing the previous concept of renal osteodystrophy, which described only the bone pathology in patients with CKD, stressing the systemic effects, including cardiovascular implications (2).

Mineral metabolism exerts a more profound influence in children with CKD as the development and maturation of the skeletal and vascular systems occur during childhood. CKD-MBD in children causes bone deformity and growth failure and may be accompanied by extra-skeletal cardiovascular calcifications with serious outcomes (3–5). Thus, monitoring and managing abnormal mineral metabolism from childhood throughout life is crucial. Although many studies have investigated CKD-MBD in adult patients, investigations solely into pediatric CKD-MBD are lacking. Studies from the United States conducted with the CKD in Children (CKiD) cohort reported the prevalence of abnormal mineral metabolism. They described fibroblast growth factor 23 (FGF-23) as the first factor to rise, followed by hyperphosphatemia and hyperparathyroidism across the CKD stages (6, 7). While their findings were comparable with the known adult studies regarding the prevalence of MBD, the study population mainly included Caucasian, African–American, and Hispanic ethinicities and the analysis was cross-sectional. Some studies have evaluated the bone markers and fracture risk in pediatric CKD patients according to race and ethnicity, showing African–American or Hispanic patients with lower 25-hidroxy vitamin D levels and a lower risk of fracture compared with Caucasian patients, although the data may be inaccurate and subjective because self-reporting was used to determine fracture history (8). To the best of our knowledge, few studies in Asia have evaluated CKD-MBD in pediatric CKD, including objective measurements regarding bone mass. Park et al. recently demonstrated the overall baseline characteristics of the Korean cohort study for Outcome in patients with Pediatric CKD (KNOW-Ped CKD) cohort featuring some of the characteristics associated with CKD-MBD, including hyperphosphatemia and hyperparathyroidism, but not focusing on many other factors associated with CKD-MBD (9).

Therefore, the objectives of this study were to comprehensively understand the relationship between renal function and factors related to MBD and change in bone mass and evaluate the prevalence and distribution characteristics of MBD, specifically among Korean patients from the KNOW-PedCKD cohort.

## Materials and methods

### Study design and population

From April 2011 to April 2016, a total of 469 patients < 20 years of age with CKD stages 1–5 were enrolled in the KNOW-PedCKD study, a nationwide, 10-year prospective and observational cohort study from seven major pediatric nephrology centers in Korea. Patients undergoing kidney replacement therapy or those who had cancer or had been treated for cancer within the previous five years were excluded from this cohort. The KNOW-PedCKD study is registered at ClinicalTrials.gov with the designation number NCT02165878 (submitted on June 11, 2014) and was approved by the Institutional Review Boards of all participating centers.

The detailed study protocol for comprehensive assessment of clinical findings and structured follow-up has been reported previously (10). The authors used the baseline data when the patients were enrolled in the cohort. Clinical data was also collected, such as chronological age, bone age at baseline, serum corrected total calcium, serum phosphate, serum alkaline phosphatase (ALP), serum intact fibroblast growth factor 23 (FGF-23), serum intact parathyroid hormone (iPTH), serum vitamin D (25-hydroxyvitamin D and 1,25-dihydroxyvitamin D), urine calcium-to-creatinine ratio, fractional excretion of phosphate (FEP), and bone densitometry Z-scores, as well as the intake of calcium supplements, non-calcium phosphate binders, and active vitamin D. Among the patients initially recruited, 431 were finally included in this study; patients aged > 18 years and those with missing data of interest were excluded.

### Clinical and laboratory measurements

The estimated glomerular filtration rate (eGFR) was calculated using the bedside CKiD formula (11). According to the KDIGO guidelines, CKD was defined and staged in patients older than two years as kidney damage with normal or mildly decreased GFR (GFR ≥ 60 ml/min/1.73 m^2^) (stages 1, 2) or GFR < 60 ml/min/1.73 m^2^ (stages 3–5) lasting more than 3 months (12). For patients less than two years of age, CKD was staged as previously demonstrated by the design and baseline characteristics of the KNOW-Ped CKD cohort (9, 10). The serum-corrected total calcium was calculated as follows: serum-corrected total calcium = serum total calcium + 0.8 × (4 –serum albumin). The reference range for all parameters in normal children are as follows; corrected total calcium 8.8–9.7 mg/dl, phosphate 4.8–8.2 mg/dl (0–5th day), 3.8–6.5 mg/dl (1st–3rd year), 3.7–5.6 mg/dl (4th–11th year), 2.9–5.4 mg/dl (12th–15th year), 2.7–4.7 mg/dl (16th–19th year), alkaline phosphatase 145–420 IU/l (1st–9th year), 140–560 IU/l (10th–11th year), 105–495 IU/l (12th–13th year), 70–525 IU/l (14th–15th year), 50–260 IU/l (16th–19th year), 25-hydroxyvitamin D 30–80 ng/ml, 1,25-dihydroxyvitamin D 70–100 pg/ml (infancy), 30–50 pg/ml (childhood), 40–80 pg/ml (adolescent) (13). While the reference ranges for iPTH among adults are 10–65 pg/ml, normal reference ranges among children for iPTH are not well established. FGF-23 has no established reference range for both adult and pediatric ages. Unlike normal healthy children, in the case of CKD-MBD patients, the criteria of disordered status were used as follows according to the management target. Hypocalcemia and hypercalcemia were defined when the serum-corrected total calcium was < 8.8 mg/dl and > 9.7 mg/dl, respectively. Hyperparathyroidism was defined according to the CKD stage: serum iPTH > 70 pg/ml in CKD stages 1 through 3, serum iPTH > 110 pg/ml in CKD stage 4, and serum iPTH > 300 pg/ml in CKD stage 5. Vitamin D deficiency was defined as follows: 25-hydroxyvitamin D < 30 ng/ml and 1,25-dihydroxyvitamin D < 60 pg/ml. These cutoff values were adopted from the Kidney Disease Outcomes Quality Initiative K/DOQI practice guidelines (14). Hyperphosphatemia was defined based on the normal phosphate levels according to age: serum phosphate > 8.2 mg/dl (0–5th day), > 7.35 mg/dl (6th day–0.99th year), > 6.5 mg/dl (1st–3rd year), > 5.6 (4th–11th year), > 5.4 (12th–15th year), or > 4.7 (16th–19th year) (13). Serum intact-FGF-23 was measured using a commercially available enzyme-linked immunosorbent assay (ELISA) kit (Immutopics, San Clemente, CA, United States). The *Δ*Bone age% was calculated as follows: *Δ*Bone age % = (bone age – chronological age)/chronological age × 100. The bone densitometry Z-score was calculated based on the reference values from the study by Lim et al. (15) as demonstrated with healthy Korean children and adolescents.

### Statistical analyses

Continuous variables are presented as the mean ± standard deviation and median (range). We compared the level of each variable according to the CKD stage using the Kruskal–Wallis test. The incidence of disordered mineral metabolism and distribution of medication intake were compared using the Chi-square test or Fisher's exact test. The relationship between variables and the eGFR was analyzed using linear, inverse, and compound models. The correlation between MBD and prescribed medications for MBD was analyzed using a multivariate analysis of covariance (MANCOVA). A *P*-value < 0.05 was considered to be statistically significant. We performed all statistical analyses using SAS version 9.4 (SAS Institute Inc., Cary, NC) and IBM SPSS Statistics for Windows, version 25.0 (IBM Corp., Armonk, N.Y., United States).

## Results

### The patterns of mineral metabolism according to CKD stages

The patterns of mineral metabolism according to the CKD stages and the distribution of patients are described in Table 1. The median serum calcium level was within the normal range, regardless of the CKD stage, with no significant difference between different stages. The serum phosphate, serum iPTH, serum FGF-23, and FEP levels significantly increased (*P* < 0.001) with advanced CKD stages; the median serum phosphate level (mg/dl) was 4.60, 4.90, 5.10, and 5.80 in CKD stages 3a, 3b, 4, and 5, respectively, and the levels of iPTH, FGF-23, and FEP constantly increased as the CKD stage advanced from stage 1 to 5 (*P* < 0.001). In contrast, the levels of 1,25-dihydroxyvitamin D, urine calcium-to-creatinine ratio, and bone densitometry Z-scores decreased with advancing CKD stages; the median 1,25-dihydroxyvitamin D level decreased as the CKD stage advanced from stage 1 to 4 (*P* < 0.001). The urine calcium-to-creatinine ratio and bone densitometry Z-scores significantly decreased (*P* < 0.001) as the CKD stage advanced from stage 1 to 3. The *Δ*Bone age % consistently showed negative values across all CKD stages, with no statistically significant difference between stages.

**Table 1:** Disordered mineral metabolism in the KNOW-Ped CKD cohort according to CKD stages.

Variables	Stage 1	Stage 2	Stage 3a	Stage 3b	Stage 4	Stage 5	*P*-value
**Corrected total calcium (mg/dl)**							
Patient number	78	115	75	69	76	17	0.438
Mean ± standard deviation	9.3 ± 0.6	9.4 ± 0.7	9.3 ± 0.6	9.4 ± 0.6	9.4 ± 0.8	8.9 ± 2.0	
Median (minimum–maximum)	9.2 (8.1–11.5)	9.3 (7.7–12.7)	9.3 (7.9–10.8)	9.3 (8.1–11.1)	9.5 (7.4–11.4)	9.6 (3.9–10.4)	
**Serum phosphate (mg/dl)**							
Patient number	78	116	75	69	76	17	< 0.001
Mean ± standard deviation	4.7 ± 0.7	4.6 ± 0.9	4.6 ± 0.9	4.9 ± 1.0	5.2 ± 1.3	5.8 ± 1.2	
Median (minimum–maximum)	4.8 (3–5.9)	4.6 (2.7–7.9)	4.6 (3–7.4)	4.9 (2–6.9)	5.1 (3.3–11.8)	5.8 (4.1–8.7)	
**Serum alkaline phosphatase (IU/L)**							
Patient number	78	116	75	69	76	17	0.446
Mean ± standard deviation	229.0 ± 142.1	234.1 ± 158.1	246.2 ± 182.8	238.3 ± 153.5	271.2 ± 185.5	255.7 ± 101.3	
Median (minimum–maximum)	213.5 (73–1191)	208 (46–1068)	225 (39–1366)	212 (42–902)	242.5 (56–1128)	237 (34–421)	
**Serum intact parathyroid hormone (pg/ml)**							
Patient number	78	113	75	68	76	17	< 0.001
Mean ± standard deviation	32.8 ± 22.4	47.7 ± 30.1	71.1 ± 49.8	105.3 ± 98.7	201.2 ± 216.8	437.3 ± 438.2	
Median (minimum–maximum)	27 (5.8–172)	45.4 (4.8–243)	58.1 (6.5–319.8)	79 (3.3–469.1)	135.8 (3.4–1244)	305.9 (19.8–1617)	
**25-hydroxyvitamin D3 (ng/ml)**							
Patient number	74	109	71	65	73	17	0.552
Mean ± standard deviation	20.9 ± 8.3	22.4 ± 10.4	22.2 ± 12.2	23.2 ± 12.1	21.6 ± 11.4	19.1 ± 12.6	
Median (minimum–maximum)	20.3 (5.4–40.1)	21.9 (4.0–62.6)	19.3 (6–74.3)	20.6 (2.7–58.0)	19.6 (3.7–62.3)	15.1 (4.2–47.1)	
**1,25-dihydroxyvitamin D3 (pg/ml)**							
Patient number	74	106	72	65	71	15	< 0.001
Mean ± standard deviation	41.8 ± 17.4	39.7 ± 26.2	36.6 ± 13.56	29.6 ± 12.8	29.5 ± 16.7	35.4 ± 26.8	
Median (minimum–maximum)	38.6 (10.8–96.3)	33.4 (11.4–220)	34.6 (9.9–71.5)	27.4 (7.3–77.1)	26.4 (4.1–91.3)	31 (7.3–119)	
**Intact- FGF-23 (pg/ml)**							
Patient number	38	71	50	53	56	15	< 0.001
Mean ± standard deviation	31.7 ± 37.2	34.1 ± 48.5	39.6 ± 40.5	63.3 ± 88.6	107.9 ± 210.4	207.8 ± 209.0	
Median (minimum–maximum)	20.5 (1.5–166)	21.4 (1.3–288)	30.4 (1.5–238)	32.5 (0–472.5)	44.2 (1.5–1400)	105 (11.1–738.1)	
**Urine calcium-to-creatinine ratio**							
Patient number	74	115	73	66	71	15	0.001
Mean ± standard deviation	0.09 ± 0.1	0.08 ± 0.14	0.04 ± 0.05	0.04 ± 0.08	0.05 ± 0.07	0.06 ± 0.06	
Median (minimum–maximum)	0.05 (0–0.61)	0.03 (0–0.8)	0.02 (0–0.2)	0.02 (0–0.57)	0.02 (0–0.42)	0.05 (0–0.2)	
**Fractional excretion of phosphate**							
Patient number	74	114	74	65	72	15	< 0.001
Mean ± standard deviation	0.07 ± 0.04	0.12 ± 0.07	0.17 ± 0.09	0.25 ± 0.1	0.38 ± 0.25	0.53 ± 0.27	
Median (minimum–maximum)	0.07 (0–0.22)	0.11 (0–0.52)	0.15 (0.04–0.46)	0.25 (0.05–0.61)	0.35 (0.04–1.75)	0.52 (0.15–1.03)	
**Bone densitometry Z-score**							
Patient number	47	76	44	43	46	11	0.009
Mean ± standard deviation	−0.43 ± 0.85	−0.33 ± 1.25	−0.98 ± 0.82	−0.85 ± 1.36	−0.79 ± 1.19	−0.16 ± 1.03	
Median (minimum–maximum)	−0.4 (−2.5–1.3)	−0.5 (−3.1–4.6)	−1.15 (−2.7–1.7)	−1 (−4.7–1.8)	−0.8 (−3.6–2)	0.1 (−1.6–1.3)	
**△Bone age %**							
Patient number	67	103	66	62	63	15	0.224
Mean ± standard deviation	−8.2 ± 19.3	−4.6 ± 19.6	−7.6 ± 14.8	−11.6 ± 16.4	−7.28 ± 14.76	−9.9 ± 16.4	
Median (minimum–maximum)	−5.0 (−59.5–33.3)	−3.4 (−70–118.2)	−3.4 (−57.1–22.3)	−7.8 (−70.6–15.4)	−4.7 (−55.1–16.8)	−7.8 (−44.2–20)	

The statistical significance level was set at *P* < 0.05.

### The prevalence of disordered mineral metabolism according to CKD stages

The prevalence of disordered mineral metabolism, including hypocalcemia, hypercalcemia, hyperphosphatemia, hyperparathyroidism, vitamin D deficiency, and prescribed medication according to the CKD stages, is presented in Table 2. Abnormal serum calcium levels were categorized into hypocalcemia and hypercalcemia for separate analyses. There was a tendency for an increasing prevalence of hypercalcemia in patients with advanced CKD stages (16.67%, 24.35%, 21.33%, 20.29%, 31.58%, and 47.06% at CKD stages 1, 2, 3a, 3b, 4, and 5, respectively; *P* = 0.0627), although not statistically significant. Likewise, there was a tendency for an increasing prevalence of hypocalcemia in patients with advanced CKD stages (17.95%, 15.65%, 13.33%, 13.04%, 21.05%, and 29.41% at CKD stages 1, 2, 3a, 3b, 4, and 5, respectively; *P* = 0.4863), although to a lesser extent than hypercalcemia. Furthermore, the prevalence of hyperphosphatemia and hyperparathyroidism significantly increased (*P* < 0.001) with CKD stage (hyperphosphatemia: 2.56%, 6.90%, 9.33%, 17.39%, 23.68%, and 41.18% across CKD stages 1–5, respectively, and hyperparathyroidism: 6.41%, 13.27%, 37.33%, and 57.35% across CKD stages 1–3b, respectively).

**Table 2:** Prevalence of disordered mineral metabolism in the KNOW-Ped CKD cohort according to CKD stages.

Laboratory findings	Stage 1	Stage 2	Stage 3a	Stage 3b	Stage 4	Stage 5	*P*-value
**Hypocalcemia**	14/78 (18.0%)	18/115 (15.7%)	10/75 (13.3%)	9/69 (13.0%)	16/76 (21.1%)	5/17 (29.4%)	0.486
**Hypercalcemia**	13/78 (16.7%)	28/115 (24.4%)	16/75 (21.3%)	14/69 (20.3%)	24/76 (31.6%)	8/17 (47.1%)	0.063
**Hyperphosphatemia**	2/78 (2.6%)	8/116 (6.9%)	7/75 (9.3%)	12/69 (17.4%)	18/76 (23.7%)	7/17 (41.2%)	< 0.001
**Hyperparathyroidism**	5/78 (6.4%)	15/113 (13.3%)	28/75 (37.3%)	39/68 (57.4%)	42/76 (55.3%)	9/17 (52.9%)	< 0.001
**25 (OH)vitamin D deficiency**	64/74 (86.5%)	88/109 (80.7%)	56/71 (78.9%)	48/65 (73.9%)	60/73 (82.2%)	14/17 (82.4%)	0.573
**1,25 (OH)_2_vitamin D deficiency**	63/74 (85.1%)	97/106 (91.5%)	68/72 (94.4%)	64/65 (98.5%)	66/71 (93.0%)	14/15 (93.3%)	0.092

The statistical significance level was set at *P* < 0.05.

### Use of medications according to CKD stages and their impact

The use of medications for CKD-MBD according to the CKD stage is shown in Table 3. The prevalence of calcium supplements (4.0%, 39.13%, 42.11%, and 82.35% among the CKD stages 3a, 3b, 4, and 5, respectively) and non-calcium phosphate binder (sevelamer or lamthanum carbonate) prescriptions (4.0%, 39.13%, 43.42%, and 82.35% among the CKD stages 3a, 3b, 4, and 5, respectively) showed a significant increase according to the CKD stages (*P* < 0.001). The prevalence of active vitamin D supplements (alfacalcidiol or calcitriol) also showed significant increase (*P* < 0.001) through all CKD stages (0.0%, 4.27%, 10.67%, 21.74%, 44.74%, and 64.71% among CKD stages 1–5, respectively). Multivariate analysis of covariance (MANCOVA) was performed to determine whether there was a statistically significant difference between the patients who received medications (calcium and non-calcium phosphorus binder and active vitamin D supplements) and non-users on CKD-MBD variables controlling for GFR (Table 4). Patients taking phosphorus binders had a significantly higher serum iPTH level than non-users. Those taking active vitamin D supplements showed a higher serum calcium level and a lower phosphorus level than those not taking them.

**Table 3:** Medications for CKD-MBD in the KNOW-Ped CKD cohort according to CKD stages.

Medications	Stage 1	Stage 2	Stage 3a	Stage 3b	Stage 4	Stage 5	*P*-value
**Calcium supplement**	4/78 (5.1%)	9/117 (7.7%)	3/75 (4.0%)	27/69 (39.1%)	32/76 (42.1%)	14/17 (82.4%)	< 0.001
**Active vitamin D**	0/78 (0.00%)	5/117 (4.3%)	8/75 (10.7%)	15/69 (21.7%)	34/76 (44.7%)	11/17 (64.741%)	< 0.001
**Phosphate binder**	4/78 (5.1%)	9/117 (7.7%)	3/75 (4.0%)	27/69 (39.1%)	33/76 (43.4%)	14/17 (82.4%)	< 0.001

**Table 4:** MANCOVA to determine the difference between the patients with and without medications on CKD-MBD variables.

**CKD-MBD variables**	**Phosphorus binder**		**F**	***P*-value**	**Partial *η*2**
	User (*n* = 92)	Non-user (*n* = 347)			
Corrected Calcium (mg/dl)*	9.4 ± 0.9	9.3 ± 0.7	0.581	0.446	0.001
Phosphorus (mg/dl)*	5.1 ± 1.3	4.7 ± 0.9	2.230	0.136	0.005
Intact parathyroid hormone (pg/ml)*	189.0 ± 237.2	77.0 ± 115.0	10.689	0.001	0.024
**CKD-MBD variables**	**Active vitamin D**		**F**	***P*-value**	**Partial η2**
	User (*n* = 81)	Non-user (*n* = 358)			
Corrected Calcium (mg/dl)*	9.5 ± 0.6	9.3 ± 0.7	5.104	0.024	0.012
Phosphorus (mg/dl)*	4.7 ± 0.9	4.8 ± 1.0	6.718	0.010	0.015
Intact parathyroid hormone (pg/ml)*	112.6 ± 97.7	130.4 ± 160.8	1.388	0.239	0.003

* eGFR was controlled for CKD-MBD variables.

The statistical significance level was set at *P* < 0.05.

### Relationship between the parameters of MBD and eGFR

**Figure 1:**
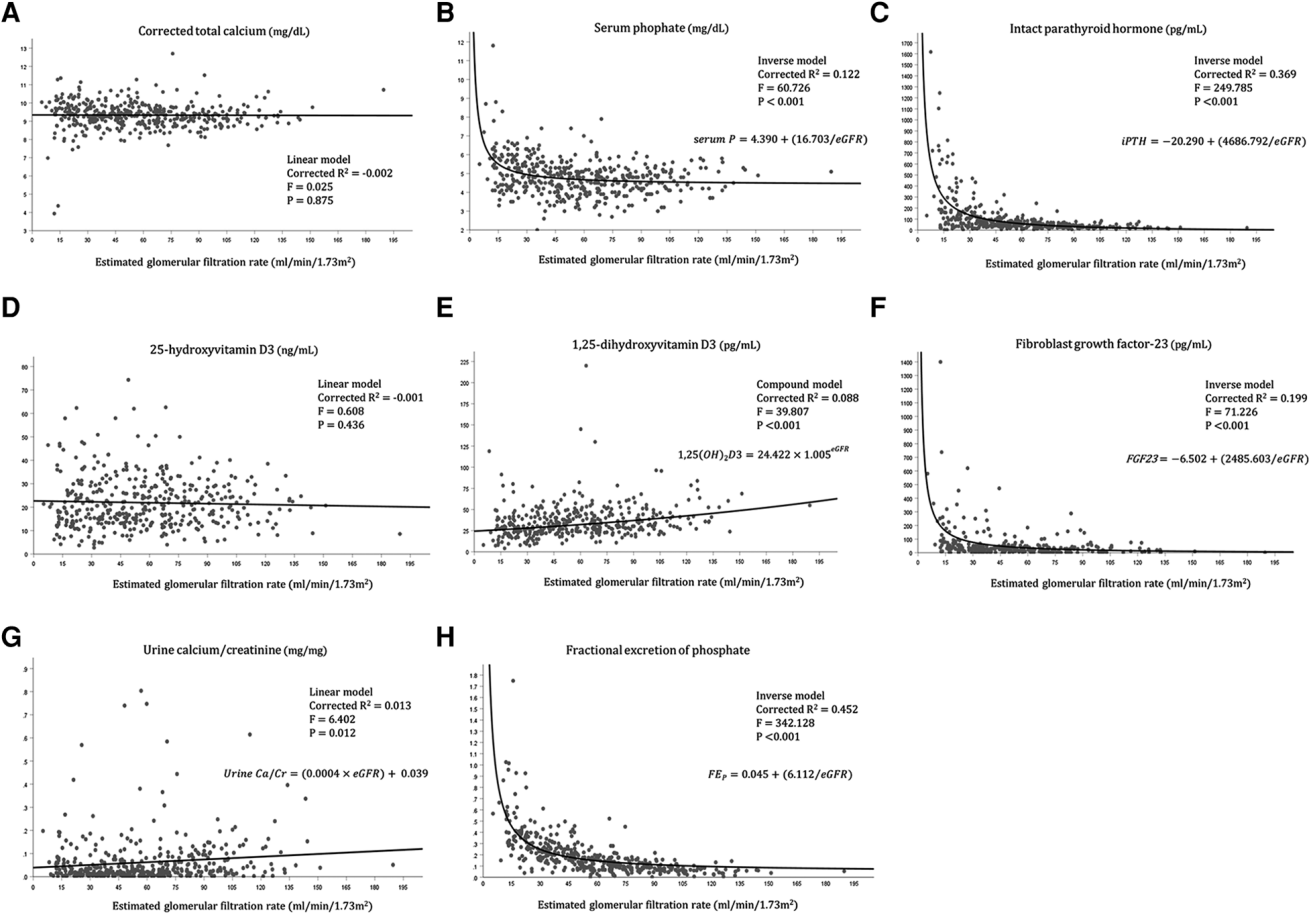
Relationship between each variable of mineral metabolism and the eGFR. (**A**) Relationship between corrected calcium level and eGFR, (**B**) Relationship between serum phosphate level and eGFR, (**C**) Relationship between intact parathyroid hormone level and eGFR, (**D**) Relationship between 25-hydroxyvitamin D3 level and eGFR, (**E**) Relationship between 1,25-dihydroxyvitamin D3 level and eGFR, (**F**) Relationship between fibroblast growth factor-23 and eGFR, (**G**) Relationship between Urine calcium/creatinine ratio and eGFR, (**H**) Relationship between fractional excretion of phosphate and eGFR.

We further investigated the relationship between each variable of mineral metabolism and the eGFR (Figure 1). The urine calcium-to-creatinine ratio and eGFR showed a significant linear association (corrected *R*2 = 0.013, *F* = 6.402, *P* = 0.012), while the linear associations between corrected total calcium level and eGFR, and between 25-hydroxyvitamin D3 and eGFR were not significant. Moreover, 1,25-dihydroxyvitamin D showed a statistically significant exponential relationship with the eGFR (corrected *R*2 = 0.088, *F* = 39.807, *P* < 0.001). Serum phosphate, serum iPTH, FGF23, and FEP showed statistically significant inverse relationships.

## Discussion

MBD is a crucial complication that should be closely monitored in pediatric patients with CKD. This study first demonstrated the prevalence of disordered mineral metabolism and its relationship with the eGFR in Korean pediatric patients with CKD for the first time in Asia.

The median serum calcium concentration remained relatively normal regardless of the CKD stage (Table 1). The stable serum calcium level until the eGFR fell below 20 ml/min/1.73 m^2^ (Figure 1) was in accordance with the course of serum calcium level in a previous study on adults with CKD (16). However, looking at the proportion of patients with hypercalcemia and hypocalcemia separately, both show an increasing tendency across the CKD stages, which is more predominant in those with hypercalcemia (Table 1). This finding was in accordance with the findings from adult CKD, in which hypocalcemia does not appear until an advanced stage of 4–5, at the expense of high PTH levels from stage 3. The increasing prevalence of hypercalcemia might be attributed to the increasing prescription of calcium supplements and active vitamin D in patients, or to tertiary hyperparathyroidism in long-standing CKD patients. In this regard, the recent 2017 KDIGO guidelines highlighting caution against hypercalcemia have profound significance due to the accumulating evidence suggesting that hypercalcemia is associated with increased mortality and nonfatal cardiovascular events (17–19). Therefore, maintaining serum calcium levels within the normal range for adults is being emphasized in clinical practice. However, owing to the lack of controlled studies and high-grade guidelines for pediatric CKD patients, and due to the negative effect on bone mass in children and adolescents, the strict criteria that are cautious in adults seem to be applied a little weakly in children to maintain calcium levels within the normal range in the age group. In this context, analysis of the follow-up data of our study might aid in evaluating calcium control in pediatric patients with CKD.

The inverse relationship between the serum phosphate level and eGFR was highlighted in our study cohort. The rapid rise in the serum phosphate level in stage 4–5 CKD (Table 1, Figure 1) was earlier than shown in previous adult CKD data from the SEEK study, while the CKiD cohort showed relatively stable phosphate levels until the eGFR fell to < 20 ml/min/1.73 m^2^ (6, 16). Higher median phosphate levels in earlier CKD stages might be associated with differences in age distribution, leading to the baseline difference in normal phosphate levels and dietary consumption. In our study, the phosphate binder prescription increased according to the increasing CKD stage, as did the rising serum phosphate level across the CKD stages. However, the lack of demonstrated efficacy of phosphate binders for lowering serum phosphate in CKD stages 3a–4, the unproven safety of phosphate binders, and the uncertainty regarding the improvement in prognosis with dietary phosphate restriction in adult studies led the treatment goal to change from “maintaining phosphate within a normal range” to “treating patients with progressive hyperphosphatemia” (19–22). This revised recommendation may also have significance in children with CKD regarding the management target of the serum phosphate level and dietary modification. Although prospective studies with pediatric patients with CKD are lacking, the choice of phosphate binders and the level of dietary restriction should be prudently titrated in pediatric patients based on the trend of phosphate levels during follow-up.

The significant increase in the iPTH level with increasing CKD stage was especially remarkable among advanced CKD stages (CKD stage 4–5) (Table 1, Figure 1), corresponding with the established adult data of hyperparathyroidism in CKD stage 3–5 (16, 23). However, the prevalence of hyperparathyroidism increased with CKD stages 1 through 3b and remained stable throughout CKD stages 4 and 5 (Table 2). This result may be explained by the increase in the reference range of the serum iPTH level in advanced CKD stages and the increased administration rate of active vitamin D supplements according to increasing CKD stages. While Japan, a neighboring nation from Asia, sets the iPTH target level slightly lower at 60–240 pg/ml and adjusts it strictly, the Korean society aims to control iPTH based on the target suggested by the K/DOQI guidelines that are universally applied to both adults and children (14, 24). Because active vitamin D, phosphate binders, and calcimimetics are relatively accessible in Korea with reimbursement, the results from this study regarding the iPTH trend can be generally applied worldwide.

The serum 25-hydroxy vitamin D deficiency and serum 1,25-dihydroxy vitamin D deficiency remained high regardless of the CKD stage (> 70.0% in all stages) (Table 1), and only the serum 1,25-dihydroxy vitamin D level showed a significant decrement with increasing CKD stage in an exponential manner. This phenomenon may be caused by a decrease in the degree of 1-*α*-hydroxylation of 25-hydroxy vitamin D owing to progressive damage to the proximal renal tubule with advancing renal deterioration, leading to a decline in the 1,25-dihydroxy vitamin D level (25). In contrast, the median level of 1,25-dihydroxy vitamin D showed a slightly higher value in stage 5 than in stage 4 CKD. This slight rebound of the 1,25-dihydroxy vitamin D level at CKD stage 5 from our study was in agreement with the results from the CKid study (6), where the 1,25-dihydroxy vitamin D level significantly decreased with decreasing eGFR but slightly increased with an eGFR < 20 ml/min/1.73 m^2^. These findings can be explained by the higher prescription rate of active vitamin D supplements in patients with an eGFR < 20 ml/min/1.73 m^2^ and those with CKD stage 5 in the CKid and our study.

MANCOVA results showing the correlation between phosphorus binders or vitamin D and CKD-MBD variables are confusing in Table 4. In this analysis, phosphorus binders and active vitamin D did not show a significant effect on serum phosphorus and iPTH levels. These findings might be due to a limitation of this cross-sectional study, which only reveals simultaneous clinical situations without defining a time sequence between the medication and laboratory findings. For example, when patients with hyperphosphatemia are prescribed a phosphorus binder, they might have normal phosphorus levels at the sampling time, while those with normal phosphorus levels not receiving phosphorus binders would continue to have normal phosphorus levels, thereby disturbing the causal relationship.

The increase in the FGF-23 level with the decrease in renal function observed in our study was similar to previous findings from studies with children (6, 26, 27). FGF-23 is a bone-derived circulating hormone that stimulates phosphate excretion through renal proximal tubular cells and suppresses the renal synthesis of 1,25-dihydroxy vitamin D and their level, and increases to lower the levels of serum phosphate toward a normal range, which was shown from the CKid study as the earliest detectable abnormality in mineral metabolism (6). FGF-23 secretion increases as CKD progresses, and the serum phosphate level is theoretically maintained at a relatively normal level until progression to advanced CKD (28). Our study demonstrated this mechanism well with the inverse relationship between eGFR and FGF-23 and between eGFR and FEP. However, as patients progress to advanced CKD, although the excretion of phosphate is increased from each renal unit, the overall excretion of renal phosphate diminishes due to a decline in the absolute number of functioning nephrons. Therefore, hyperphosphatemia inevitably ensues. Increasing FEP with CKD progression, as shown from our study, is a sign of nephron stress, which the physiologic adaptive mechanism has become maladaptive, leading to over-excretion of phosphate and compensation for the absolute number of nephron loss as CKD progress (29). This phenomenon was also shown from previous adult studies showing increasing FEP with decreasing creatinine clearance, leading to neutral phosphorus balance in some CKD stage 3/4 stage patients (21, 30). Several studies have reported that increased FGF-23 levels are associated with CKD progression, left ventricular hypertrophy, and premature death (31–34). Accumulating evidence suggests that FGF-23 levels are further associated with cognitive dysfunction and even host defense and inflammation (35–37). Although there are no guidelines for reducing FGF-23 levels in patients with CKD, dietary phosphate restriction, non-calcium phosphate binders, intensified dialysis, and renal transplantation seem to be reliable options in both children and adults with CKD. As active vitamin D further stimulates FGF-23 secretion, using a minimal dosage of active vitamin D to reach the target iPTH value should be emphasized in clinical practice (38).

As CKD progresses in children, more patients are at risk of decreased bone density caused by CKD-MBD. For the first time, we demonstrated the progressive deterioration in bone density as CKD progressed from stages 1 through 3 (Table 1). Although the bone density improves in CKD stages 4–5, it consistently falls behind the bone age compared to chronological age across all CKD stages among the KNOW-Ped CKD cohort. The decreasing bone density then rebounds according to the increasing CKD stage, which can be partly explained by the increasing interventions, including the intake of vitamin D, calcium, and phosphate binders. Bone densitometry has limitations as an indicator of the comprehensive status of bone disease. Because of the age distribution and different growth velocities among patients, it does not indicate the state of bone turnover, making it difficult to assess the presence of adynamic bone disease. Additionally, because it relies on areal density rather than volumetric density, the bone density of short statured children is usually underestimated (39–41). However, we believe understanding the current status of the bone density distribution has certain significance in the setting of limited bone biopsy due to its invasiveness and burden.

This study has several limitations that warrant discussion. First, missing data or unavailable information, a different number of patients for each CKD stage might have caused biased results, skewing the tendency of mineral concentration across the CKD stages. Second, the level of dietary restriction and compliance with each patient's medications, which may have impacted the serum mineral or hormone levels, were not included. Third, risk factor analysis was not conducted, nor the subgroup analysis according to different etiology was not performed as the cohort was composed of patients with heterogeneous etiologies (including glomerulopathy, congenital anomalies of kidney and urinary tract, cystic kidney disease, etc.) and progression rates. Therefore, we determined that it was unconvincing to demonstrate the association of a single factor with CKD-MBD progression since CKD “*per se*” can also influence the factor. Fourth, we did not analyze baseline cardiovascular parameters in this study due to the low prevalence of detectable extra-skeletal calcifications. Finally, owing to the multicenter study design, there may have been potentially significant inter-study variations among individual center settings. However, the strength of this study lies in that this is the first nationwide cohort study of pediatric CKD-MBD and the first prospective and most extensive pediatric CKD cohort study in Asia, demonstrating the distribution and prevalence of CKD in Asian ethnicity and presenting the relationship between eGFR change and variables associated with CKD-MBD in a pediatric population. As Korea has good access to CKD-MBD-related medications and a relatively well-established reimbursement system, the management of Korean pediatric CKD patients closely conforms to universal guidelines, and thus the results of this study may be applicable worldwide. Therefore, the baseline study of mineral metabolism is meaningful as a cornerstone of future research. We will follow the study participants for ten years. We expect further studies regarding dynamic changes in mineral metabolism and growth, including analysis by underlying etiologies in children with CKD from the full-length cohort data to be available.

## Conclusion

Among Korean pediatric patients from the baseline data of the KNOW-PedCKD cohort, the median calcium level remained relatively normal with increasing use of calcium supplements and active vitamin D across the CKD stages. Disordered mineral bone metabolism with respect to serum phosphate, serum FGF-23, hyperparathyroidism, and bone densitometry Z-score was evident with advancing CKD stages. Further study with follow-up data showing the trajectory or course of CKD-MBD stratified with underlying CKD etiologies might elucidate the progression and the impact of medical management on CKD-MBD in pediatric patients. Furthermore, a comparative study and consensus on what modality should be used for assessment of bone disease, growth, and extra-skeletal calcification are needed.

## Data Availability

The datasets generated for this study is accessible after approval by the investigators of KNOWPed-CKD study (http://www.know-pedckd.org/pedckd/main/main.html). To request datasets, contact the corresponding author.
