# Corrigendum: Mineral bone disorder in children with chronic kidney disease: data from the KNOW-Ped CKD (Korean cohort study for outcome in patients with pediatric chronic kidney disease) study

**DOI:** 10.3389/fped.2023.1221302

**Published:** 2023-06-14

**Authors:** Jiwon Jung, Keum Hwa Lee, Eujin Park, Young Seo Park, Hee Gyung Kang, Yo Han Ahn, Il-Soo Ha, Seong Heon Kim, Heeyeon Cho, Kyoung Hee Han, Min Hyun Cho, Hyun Jin Choi, Joo Hoon Lee, Jae Il Shin

**Affiliations:** ^1^ Department of Pediatrics, Asan Medical Center Children’s Hospital, Ulsan University, College of Medicine, Seoul, Republic of Korea; ^2^ Department of Pediatrics, Severance Children’s Hospital, College of Medicine, University of Yonsei, Seoul, Republic of Korea; ^3^ Department of Pediatrics, Hallym University Kangnam Sacred Heart Hospital, Seoul, Republic of Korea; ^4^ Department of Pediatrics, Seoul National University Children’s Hospital, College of Medicine, Seoul National University, Seoul, Republic of Korea; ^5^ Department of Pediatrics, Samsung Medical Center, School of Medicine, Sungkyunkwan University, Seoul, Republic of Korea; ^6^ Department of Pediatrics, School of Medicine, Jeju National University, Jeju, Republic of Korea; ^7^ Department of Pediatrics, School of Medicine, Kyungpook National University, Daegu, Republic of Korea; ^8^ National Institute of Food and Drug Safety Evaluation, Ministry of Food and Drug Safety, Cheongju, Republic of Korea; ^9^ Institute of Kidney Disease Research, College of Medicine, Yonsei University, Seoul, Republic of Korea

**Keywords:** mineral bone disorder, hyperphosphatemia, hyperparathyroidism, chronic kidney disease, bone densitometry

A Corrigendum on Mineral bone disorder in children with chronic kidney disease: Data from the KNOW-Ped CKD (Korean cohort study for outcome in patients with pediatric chronic kidney disease) study By Jung J, Lee KH, Park E, Park YS, Kang HG, Ahn YH, Ha I, Kim SH, Cho H, Han KH, Cho MH, Choi HJ, Lee JH and Shin JI. (2023) Front. Pediatr. 11:994979. doi: 10.3389/fped.2023.994979

**Incorrect Funding**

In the published article, there was an error in the Funding statement. This work was funded by grants from Korea Centers for Disease Control and Prevention after peer review (fund codes: 2011E3300300, 2012E3301100, 2013E3301600, 2013E3301601, 2013E3301602, 2016E3300200, 2016E3300201, 2016E300202, 2019E320100, 2019E320101, 2019E320102, and 2022-11-007). The correct Funding statement appears below.

**FUNDING**

This work was funded by grants from the Korea Disease Control and Prevention Agency (fund codes: 2011E3300300, 2012E3301100, 2013E3301600, 2013E3301601, 2013E3301602, 2016E3300200, 2016E3300201, 2016E300202, 2019E320100, 2019E320101, 2019E320102, and 2022-11-007).

The authors apologize for this error and state that this does not change the scientific conclusions of the article in any way. The original article has been updated.

